# Growth Characteristics of *Adenophora triphylla* var. *japonica* Hara Seedlings as Affected by Growing Medium

**DOI:** 10.3390/plants8110466

**Published:** 2019-10-31

**Authors:** Hye Ri Lee, Hyeon Min Kim, Hyeon Woo Jeong, Gam Gon Kim, Chae In Na, Myung Min Oh, Seung Jae Hwang

**Affiliations:** 1 Division of Applied Life Science, Graduate School of Gyeongsang National University, Jinju 52828, Korea; dgpfl77@naver.com (H.R.L.); s75364@daum.net (H.M.K.); J_dk94@naver.com (H.W.J.); rkarhs0106@gmail.com (G.G.K.); nachaein@gnu.ac.kr (C.I.N.); 2 Department of Agricultural Plant Science, College of Agriculture & Life Science, Gyeongsang National University, Jinju 52828, Korea; 3 Division of Animal, Horticulture and Food Sciences, Chungbuk National University, Cheongju 28644, Korea; moh@cbnu.ac.kr; 4 Institute of Agriculture & Life Science, Gyeongsang National University, Jinju 52828, Korea; 5 Research Institute of Life Science, Gyeongsang National University, Jinju 52828, Korea

**Keywords:** root surface area, root volume, terra-plug

## Abstract

*Adenophora triphylla* var. *japonica* Hara is a highly valued medicinal plant that is used to treat or prevent bronchitis, cough, cancer, and obesity. However, there has been no study on the production of *Adenophora triphylla* var. *japonica* Hara seedlings in a closed-type plant production system (CPPS). This study was conducted to examine the growth characteristics of *Adenophora triphylla* var. *japonica* Hara seedlings as affected by different growing media. The seeds were sown on a 128-cell plug tray filled with urethane sponges (US), LC grow foam (LC), rockwool (RW), or terra-plugs (TP). The seedlings were cultured for a duration of 54 days under temperature 25 ± 1°C, a photoperiod of 12/12 h (light/dark), and light intensity of 180 µmol·m^−2^·s^−1^ photosynthetic photon flux density provided by RB LEDs (red:blue = 8:2) in a closed-type plant production system (CPPS). The germination rate of *Adenophora triphylla* var. *japonica* Hara was significantly highest in the TP. Also, seedling shoot growth indicators of plant height, leaf length, leaf width, number of leaves, fresh weight (FW), and dry weight (DW) of the shoot, and leaf area were markedly the greatest in the TP and the lowest in the US. The SPAD (soil-plant analysis development) value was higher in the TP and US than in the LC or RW. In addition, the seedling root growth characteristics of total root length, root surface area, root volume, and number of root tips were significantly greatest in the TP. Moreover, the maximum root diameter, FW and DW of roots were the greatest in the TP. In conclusion, the results suggest that TP are viable for the growth development of *Adenophora triphylla* var. *japonica* Hara seedlings.

## 1. Introduction

Medicinal crops are being developed not only as traditional herbal medicines but also as raw material for high-value industries, and they are being expanded to natural materials such as medicine, functional foods, cosmetics, and daily supplies. In particular, the culture area of medicinal crops has been expanded because the market for functional food and medicinal crops is expanding with the increasing interest in health [1,2]. However, for many medicinal crops, there is inadequate clear information on environmental conditions such as seed germination, seedling, and culture after transplanting. Moreover, since most of the medicinal crops are grown in a natural environment, there are concerns associated with biotic or abiotic contamination, adulteration of species and weeds, and quality variation of the crop products themselves. These problems can be solved using a closed-type plant production system (CPPS) which can be artificially controlled.

Growing media (peat, sand, gravel, polyurethane foam, expanded polystyrene, geotextiles, perlite, rockwool, vermiculite, coir, etc.) have long been used for growing ornamental and horticultural plants in different forms and sizes of container [3,4]. In a CPPS, the entire process from sowing to harvesting takes place inside. Therefore, in order to ensure a stable production of crops, a proper growing medium should be used for sowing, seedling, and transplanting, and the rhizosphere environment should be maintained in a suitable state during the cultivation period. That is, plant support, sufficient gas exchange as well as a stable pH should be maintained, and an appropriate nutrient solution level and composition should be made. Artificial media such as urethane sponges or rockwool, which easy to use in hydroponic culture and with less particles and dust of the medium, are used in a CPPS. Although urethane sponges have the advantage of being cheap and easy to handle, the physical structure of the urethane sponge causes vertical water movement [5,6]. Therefore, it is difficult to keep much moisture, such that the reported germination rate is low because the upper part of the growing medium dries out. Rockwool is uniform in its physical properties and moisture is easily managed, but it is a cause of raised production costs of crops due to its high import cost, and it is also difficult to dispose of after use and causes environmental pollution. To solve this problem, some agricultural companies and researchers who professionally produce plant growing medium are studying the development and application of stable media that are environmentally friendly, low cost, and stable to supply. Newly developed medium (LC and TP) are expected to improve the uniformity, absorbency, and economy of growing mediums. In addition, physical property (total porosity, container capacity, and bulk density) and chemical property (pH, electrical conductivity, cation exchange capacity, and nutrient) can be controlled during the production process. Also, germination and growth are affected by the growing media [7]. The physical properties and nutrients of the growing medium can be controlled during the production process, so that a growing medium with proper physical properties and nutrients can be selected according to the crop.

*Adenophora triphylla* var. *japonica* Hara is a perennial plant of the genus *Campanulaceae*, with more than ten species such as *Adenophora triphylla* var. hirsute, *Adenophora triphylla* A. radiotifolia, and *Adenophora stricta.* It is mainly found in Korea, China, Japan, and Russia, and is grown in sunny areas at the foot of mountains with good drainage [8,9]. Conventionally, *Adenophora triphylla* var. *japonica* Hara was used as a food to prevent obesity in Korea [10,11]. It contains different phytochemicals such as saponin, inulin, polysthicol, lupenone, triphyllol, and daucosterol [12], and anti-mutagenic and anti-tumor effects of the ethanol extract and its fractionation [13] and antioxidant effects of the ethylacetate extract of the root [14] have been reported. In addition, detoxification, anti-cancer, and anti-diabetic effects and protection of brain nerve cells have been reported, and its consumption as herbal medicine is increasing [14,15,16]. As it is a health functional food, interest is rising, and the culture of the plant is being attempted because natural harvesting cannot meet the rapidly increasing demand. However, previous studies have focused on classification, nutritional composition, and the functionality of those phytochemicals, and any previous study on cultivation techniques is insufficient.

Therefore, this study aimed to investigate the germination, growth characteristics, and morphological characteristics of shoots and roots of *Adenophora triphylla* var. *japonica* Hara in order to select a suitable growing medium for producing high-quality *Adenophora triphylla* var. *japonica* Hara seedlings in a CPPS.

## 2. Results and Discussion

### 2.1. Physicochemical Properties of the Growing Medium

The physicochemical properties of the growing medium such as the container capacity, air space, total porosity, bulk density, pH, and electric conductivity (EC) are shown in Table 1. The container capacity was highest in the LC grow foam (LC) at 85.45% and the lowest in the urethane sponges (US) at 62.56%. The container capacity is the maximum amount of water that a growing medium can hold as an indicator of the water and nutrients the growing medium may contain after draining in a natural state. A growing medium with a high container capacity has high physical buffering power and relatively easy water management [17]. On the other hand, in a growing medium with low container capacity, the root is stressed to inhibit the uptake of the water and nutrient, and growth is inhibited because the water content is kept low for a long time in the rhizosphere [18,19]. The air space is an important indicator for the use of rhizospheric oxygen. It was the greatest in the LC at 5.14%, followed by the US at 3.06%, and the rockwool (RW) at 2.48%, and it was the lowest in the terra-plugs (TP) at 1.35%. The total porosity showed a similar tendency: that of, LC was higher than that of the other growing media. The bulk density was 0.02–0.05 g∙cm^−3^ in the all growing media except TP. When the bulk density of a medium is too low, the growing medium loses its ability to support and hold the crops. On the other hand, too high a weight can increase transportation costs and reduce the total porosity, container capacity, and air space. It has been reported that the optimum physical properties are minimum total porosity 85%, container capacity between 55% and 75%, and air space between 20% and 30% for an ideal growing medium for plant growth [20]. However, we must consider not only that the amount of growing medium used for germination and seedling was very small, but also that the growing medium used in the experiment was different because it was formed growing medium produced by compressing to a certain uniform form.

The pH is a measure of the acidity of the medium determined by taking a log of the reciprocal of the hydrogen ion (H^+^) concentration [21]. The pH values of the growing media used in this study were in the range of 6.39–7.14. This is higher than the recommended pH range of 5.5–6.5 suggested by Nelson [22], but this has not been shown to have a negative impact through nutrient solution management during cultivation. The EC was the highest in the TP at 0.30 dS∙m^−1^. This result was considered to be because the TP is made of organic materials. In the case of US, LC, and RW, it was measured in the range of 0.04–0.13 dS∙m^−1^, showing the chemical characteristics of typical inorganic materials.

### 2.2. Germination Rate

**Figure 1:**
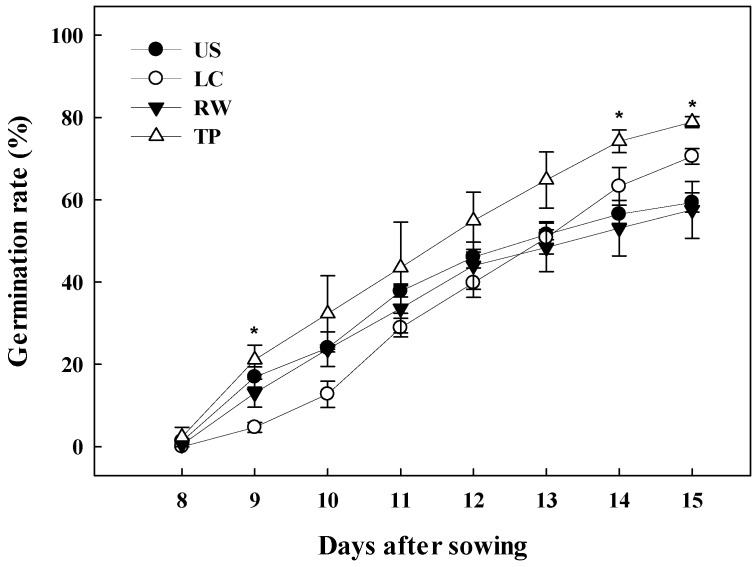
The germination rate of *Adenophora triphylla* var. *japonica* Hara as affected by different media. US, urethane sponges; LC, LC grow foam; RW, rockwool; and TP, terra-plugs. Vertical bars represent standard deviation from the mean (n = 3). * Significant at *p* ≤ 0.05.

The germination rate of *Adenophora triphylla* var. *japonica* Hara as affected by different media is shown in Figure 1. The initial germination rate was the highest in the TP at 21.09%, and the lowest in the LC at 4.69% at 9 days after sowing. Similarly, the final germination rate was highest in the TP at 78.90%, followed by the LC at 70.57%, and the lowest rates were in the US and RW at 59.38% and 57.55%, respectively. This result was attributed to the physical properties of the growing media. US and RW were considered to not have enough moisture for germination because their container capacity values were lower than those of the other growing media. Usually, urethane sponges lose more moisture rapidly than other media because the physical structure of the urethane sponge causes vertical water movement [23]. As an example, it was reported by Seo [5] that germination was the lowest in urethane sponges among rockwool, perlite, coir, and urethane sponges.

### 2.3. Growth Characterics

**Figure 2:**
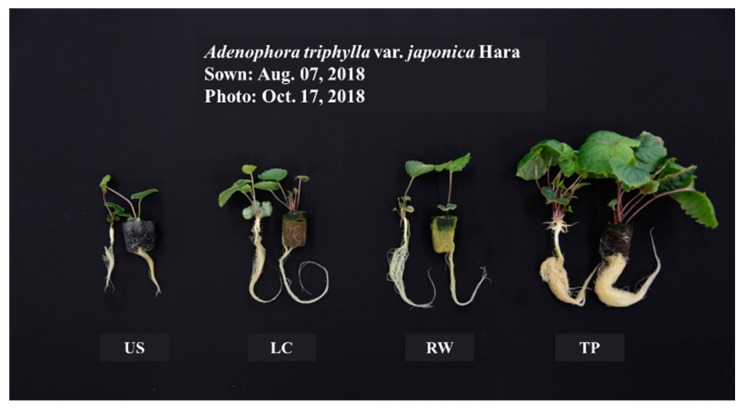
Growth of *Adenophora triphylla* var. *japonica* Hara seedlings as affected by different media at 54 days after transplanting. US, urethane sponges; LC, LC grow foam; RW, rockwool; and TP, terra-plugs.

The growth of *Adenophora triphylla* var. *japonica* Hara seedlings as affected by different media at 54 days after transplanting is shown in Table 2 and Figure 2. The plant height and leaf length were the shortest in the US at 3.97 cm and 1.42 cm, respectively. The leaf width, crown diameter, and number of leaves were the greatest in the TP at 6.07 cm, 4.97 mm, and 12.6, respectively. Also, the fresh weight (FW) and dry weight (DW) of the shoots and roots were the heaviest in the TP. The leaf area was the greatest in the TP. The soil-plant analysis development (SPAD) was highest in the US at 49.52. This result appears to be related to the EC of the medium. The EC of the TP was the highest at 0.30 dS∙m^−1^, and EC of US was the lowest at 0.04 dS∙m^−1^ (Table 1). The growth of *Adenophora triphylla* var. *japonica* Hara seedlings appeared to be superior due to the nutrient content of TP, an organic growing medium. The nutrients in the growing medium were considered to have contributed to early growth. Ions eluted from the medium affect the EC level of water in the medium and affect the plant growth [24,25,26]. This result is similar to the result of the excellent growth of tomato seedlings in organic growing media such as the Q-plug growing medium, which had a high EC level among the media used in the experiment [27]. Moreover, seedlings have a high demand for mineral nutrients, which, results in a rapid growth rate [28].

### 2.4. Root Morphology

**Figure 3:**
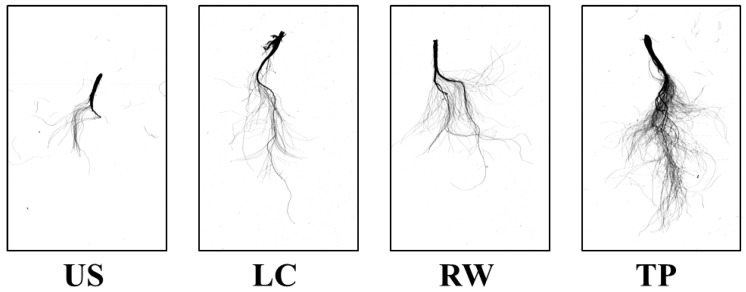
Root images of *Adenophora triphylla* var. *japonica* Hara seedlings as affected by different media at 54 days after transplanting. US, urethane sponges; LC, LC grow foam; RW, rockwool; and TP, terra-plugs.

**Figure 4:**
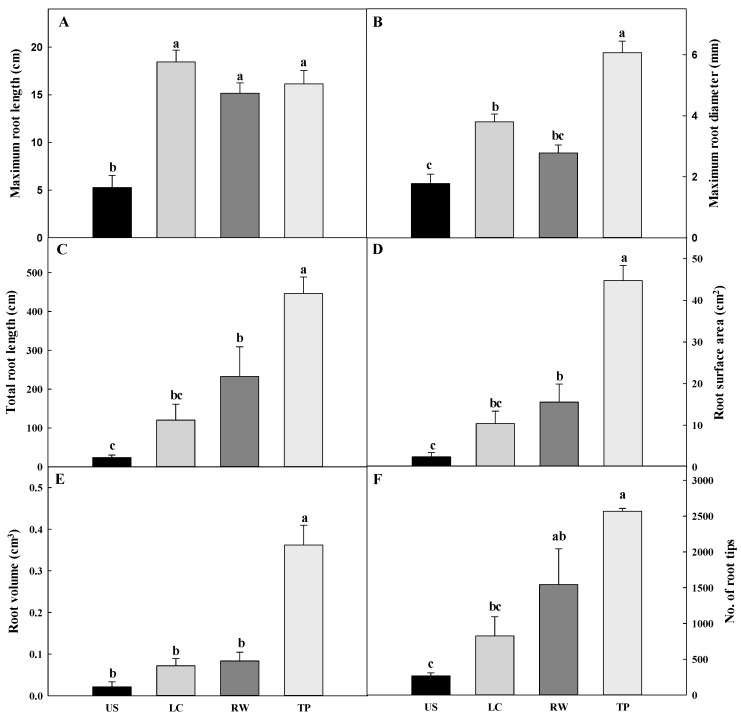
Maximum root length (**A**), maximum root diameter (**B**), total root length (C), root surface area (**D**), root volume (**E**), and number of root tips (**F**) of *Adenophora triphylla* var. *japonica* Hara seedlings as affected by different media at 54 days after transplanting. US, urethane sponges; LC, LC grow foam; RW, rockwool; and TP, terra-plugs. Vertical bar represent the standard deviation of the mean (*n* = 3). Different letters in the same column indicate significant differences based on Tukey’s multiple range test (*p* ≤ 0.05).

The root morphology of *Adenophora triphylla* var. *japonica* Hara seedlings as affected by different media at 54 days after transplanting is shown in Figure 3 and Figure 4. The maximum root length was shortest in the US at 5.29 cm, and those in the other growing media were over 15 cm. The maximum root diameter was the greatest in the TP, 3.3 times thicker than that in the US. The total root length, root surface area, root volume, and number of root tips tended to be the greatest in the TP. In particular, the total root length was 72 times longer than that in the US. The root surface area was the largest in the TP at 44.71 cm^2^, and the lowest in the US at 2.44 cm^2^. The root volume in the TP, at 0.36 cm^2^, was more than 4 times higher than those in the other growing media. Also, the number of root tips was the highest in TP at 2569, followed by RW (1542), LC (825), and US (268). Souch et al. [29] reported that this indicator is most sensitive to soil compaction because the roots are thinner at the seedling stage, which gives more resistance to growth. Tracy et al. [30] observed that primary root elongation was most rapid during the first 4 days of the experiment period, suggesting that water and nutrients are important to the newly emerged radicle for anchoring the root system and supporting initial shoot growth. This result is consistent with the EC level of the growing medium. When seedlings in other growing media were still primarily reliant on seed-derived nutrients, seedlings in TP, which had high EC, showed increased early growth. The observed improvement in growth under moderate soil compaction may have resulted from increased root to soil contact, enabling more rapid water and nutrient uptake to supply the growing plant. Also, it has been observed that moderate bulk densities enhance root growth [30,31,32].

## 3. Materials and Methods

### 3.1. Plant Material and Growth Conditions

**Figure 5:**
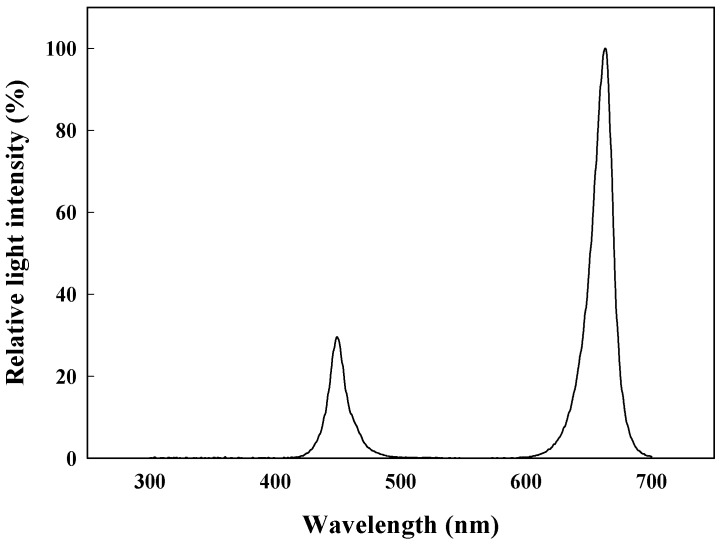
Relative spectral distribution of RB LEDs (red:blue = 8:2) used in a closed-type plant production system.

*Adenophora triphylla* var. *japonica* Hara seeds were sown in 128-cell plug trays filled with different media on August 07, 2018, and were germinated in a closed-type plant production system (CPPS, C1200H3, FC Poibe Co. Ltd., Seoul, Republic of Korea) for a duration of 15 days. Germination conditions were set to a temperature of 29 ± 1°C, 60 ± 10% relative humidity, and a photoperiod of 12/12 (light/dark) under red light emitting-diodes (LEDs, ES LEDS Co. Ltd., Seoul, Republic of Korea) with 180 ± 10 µmol∙m^−2^∙s^−1^ photosynthetic photon flux density (PPFD) set with a photometer (Hd2101.2, Delta Ohm SrL, Selvazzano Dentro, Italy). The 33 seedlings of uniform size per treatment were transplanted into a deep floating system with recycling Hoagland nutrient solution [33] (pH 5.8 and EC 1.0 dS∙m^−1^). The growth conditions were maintained at temperature 25 ± 1°C, 60% ± 10% relative humidity, and photoperiod 12/12 (light/dark) under RB LEDs (red:blue = 8:2) with 180 ± 10 µmol∙m^−2^∙s^−1^ PPFD (Figure 5) for a duration of 54 days. Nutrient solution was replaced every two weeks, and pH/ EC measurement was performed using a multiparameter (8200M, GOnDo Electronic Co., Ltd., Taipei, Taiwan).

### 3.2. Medium and Physicochemical Properties

The growing media used in this study were urethane sponges (US, Gafatec Co. Ltd., Hwaseong, Republic of Korea), LC grow foam (LC, Smithers-Oasis Co. Ltd., OH, USA), rockwool (RW, Grodan CO. Ltd., Roermond, The Netherlands), and terra-plugs (TP, Smithers-Oasis Co. Ltd., OH, USA).

To measure the physical properties such as total container capacity, air space, total porosity, and bulk density, the wet weight was measured after the growing medium was soaked for 48 h, and then the weight of the growing medium and the volume of drained water were measured after draining for 2 h at room temperature. The dry weight was measured after being completely dry for a duration of 72 h. The measured value was calculated using the formula given by Fonteno and Choi et al. [34,35].

Container capacity = [((wet weight-dry weight))⁄volume of medium] × 100(1)

Air space = (volume of drained water⁄volume of medium) × 100(2)

Total porosity = container capacity + air space(3)

Bulk density = dry weight⁄(volume of medium)(4)

To measure the chemical properties, each medium was mixed in a ratio of 1:5 (*v/v*) with distilled water and shaken using shaker (KS-500, Koencon Co. Ltd., Hanam, Republic of Korea) for 3 h, then the pH and EC were measured using a multiparameter.

### 3.3. Growth Characteristics

The germination rate of *Adenophora triphylla* var. *japonica* Hara was investigated from the 8th day to the 15th days after sowing. At 54 days after transplanting, the plant height, leaf length, leaf width, crown diameter, number of leaves, leaf area, and FW and DW of shoots and roots were measured. The chlorophyll content was represented as the SPAD, which was measured using a portable chlorophyll meter (SPAD-502, Konica Minolta Inc., Tokyo, Japan). The diameter of crown, the point where the shoot and the root meet, was measured using a vernier caliper (CD-20CPX, Mitutoyo Co. Ltd., Kawasaki, Japan). The leaf area was measured using a leaf area meter (LI-3000, LI-COR Inc., NE, USA). The FW was measured using an electronic balance (EW220-3NM, Kern & Sohn GmbH., Balingen, Germany), and the DW was measured after drying in an oven (Venticell-220, MMM Medcenter Einrichtunger GmbH., Planegg, Germany) at 70 °C for 72 h. To measure the root characteristics of the seedling, as affected by the medium, root length was measured as that of the longest root and the maximum root diameter was measured using a vernier caliper. The total root length, root surface area, root volume, number of root tips, and total root length by different root diameters were measured using the WinRhizo Pro 2007a image analysis system (Regent Instruments, Sainte-Foy Co. Ltd., QC, Canada) coupled with an Expression 1000XL professional scanner (Epson America Inc., CA, USA).

### 3.4. Statistical Analysis

A randomized complete block design with 3 replications and 11 plants in each replication. To determine the effect of growing medium, 9 plants per treatment were used to determine growth characteristics. To analyze the growing medium physicochemical properties and root morphology, 3 replicates were used for each growing medium. The statistical analysis was carried out using the Statistical Analysis System program (SAS 9.1, SAS Institute Inc., NC, USA). The experiment results were subjected to analysis of variance (ANOVA) and Tukey’s multiple range tests. Graphing was performed with the SigmaPlot program (SigmaPlot 12.0, Systat Software Inc., CA, USA).

## 4. Conclusions

The present study indicated that the growing medium significantly affected the growth of *Adenophora triphylla* var. *japonica* Hara seedlings. The germination rate showed a great increase in TP, which had a higher EC level than the other growing media. The growth of *Adenophora triphylla* var. *japonica* Hara seedlings measured in term of the plant height, FW and DW of shoots and roots, and leaf area was greatly enhanced in TP in a CPPS. There were significant differences affected by the growing medium in aspects of the root morphology such as length, diameter, surface area, volume, and number of root tips. In conclusion, early seedling growth and root development were good, and it is also confirmed that the availability of TP growing medium as an alternative medium solves the problem of cost and the disposal method. Additional study is needed on adding nutrients to other growing media.

## Figures and Tables

**Table 1:** The physicochemical properties of the growing media used in the experiment (n = 3).

Medium ^z^	Container Capacity (%)	Air Space (%)	Total Porosity (%)	Bulk Density (g∙cm^−3^)	pH	EC ^y^ (dS∙m^−1^)
US	62.56 b ^y^	3.06 ab	65.62 b	0.02 b	6.61 bc	0.04 c
LC	85.45 a	5.14 a	90.59 a	0.02 b	7.14 a	0.13 b
RW	66.23 ab	2.48 ab	68.71 b	0.05 b	6.80 b	0.10 b
TP	77.87 ab	1.35 b	79.23 b	0.12 a	6.39 c	0.30 a

^z^ US, urethane sponges; LC, LC grow foam; RW, rockwool; and TP, terra-plugs. ^y^ Mean separation within columns by Tukey’s multiple range at *p* ≤ 0.05.

**Table 2:** The growth characteristics of *Adenophora triphylla* var. *japonica* Hara seedlings as affected by different media at 54 days after transplanting (n = 3).

Medium ^z^	Plant Height (cm)	Leaf Length (cm)	Leaf Width (cm)	Crown Diameter (mm)	No. of Leaves	Fresh Weight (g/plant)		Dry Weight (g/plant)		Leaf Area (cm^2^/plant)	SPAD Value
						Shoot	Root	Shoot	Root		
US	3.97 c^y^	1.42 b	1.97 c	1.39 b	4.1 b	0.12 b	0.13 b	0.018 b	0.014 b	8.51 b	49.52 a
LC	8.20 ab	2.36 a	4.30 b	2.54 b	8.2 ab	0.62 b	0.72 ab	0.107 b	0.070 b	26.38 b	42.03 bc
RW	7.48 b	2.13 ab	3.34 bc	1.87 b	6.6 b	0.38 b	0.46 b	0.057 b	0.045 b	16.15 b	37.69 b
TP	10.51 a	2.74 a	6.07 a	4.97 a	12.6 a	2.34 a	1.75 a	0.370 a	0.209 a	62.43 a	47.43 a
Significance	***	**	***	***	***	***	***	**	***	***	***

^z^ US, urethane sponges; LC, LC grow foam; RW, rockwool; and TP, terra-plugs. ^y^ Mean separation within columns by Tukey’s multiple range at *p* ≤ 0.05. ^**, ***^ Significant at *p* ≤ 0.01, 0.001, respectively.
